# Quantitative Analysis of the Cardiac Phosphoproteome in Response to Acute β-Adrenergic Receptor Stimulation In Vivo

**DOI:** 10.3390/ijms222212584

**Published:** 2021-11-22

**Authors:** Alican Güran, Yanlong Ji, Pan Fang, Kuan-Ting Pan, Henning Urlaub, Metin Avkiran, Christof Lenz

**Affiliations:** 1 British Heart Foundation Centre of Research Excellence, School of Cardiovascular Medicine and Sciences, King’s College London, St Thomas’ Hospital, Westminster Bridge Road, London SE1 7EH, UK; alican.guran@kcl.ac.uk (A.G.); metin.avkiran@kcl.ac.uk (M.A.); 2 Bioanalytical Mass Spectrometry Group, Max Planck Institute for Biophysical Chemistry, 37077 Goettingen, Germany; yanlong.ji@mpibpc.mpg.de (Y.J.); pan.fang@mpibpc.mpg.de (P.F.); Kuan-Ting.Pan@kgu.de (K.-T.P.); hurlaub@gwdg.de (H.U.); 3 Hematology/Oncology, Department of Medicine II, Johann Wolfgang Goethe University, 60590 Frankfurt am Main, Germany; 4 Frankfurt Cancer Institute, Johann Wolfgang Goethe University, 60590 Frankfurt am Main, Germany; 5 Department of Biochemistry and Molecular Biology, Soochow University Medical College, Suzhou 215123, China; 6 Department of Clinical Chemistry, University Medical Center Goettingen, 37075 Goettingen, Germany

**Keywords:** phosphorylation, cell signalling, mass spectrometry, β-adrenergic receptor, SILAC

## Abstract

β-adrenergic receptor (β-AR) stimulation represents a major mechanism of modulating cardiac output. In spite of its fundamental importance, its molecular basis on the level of cell signalling has not been characterised in detail yet. We employed mass spectrometry-based proteome and phosphoproteome analysis using SuperSILAC (spike-in stable isotope labelling by amino acids in cell culture) standardization to generate a comprehensive map of acute phosphoproteome changes in mice upon administration of isoprenaline (ISO), a synthetic β-AR agonist that targets both β1-AR and β2-AR subtypes. Our data describe 8597 quantitated phosphopeptides corresponding to 10,164 known and novel phospho-events from 2975 proteins. In total, 197 of these phospho-events showed significantly altered phosphorylation, indicating an intricate signalling network activated in response to β-AR stimulation. In addition, we unexpectedly detected significant cardiac expression and ISO-induced fragmentation of junctophilin-1, a junctophilin isoform hitherto only thought to be expressed in skeletal muscle. Data are available via ProteomeXchange with identifier PXD025569.

## 1. Introduction

β-adrenergic receptor (β-AR) stimulation represents a powerful mechanism for acutely enhancing cardiac output, which is a central component of the fight-or-flight response [1]. Activation of stimulatory G (Gs) protein-coupled β-ARs leads to increased activity of adenylate cyclase and subsequent production of cyclic adenosine monophosphate (cAMP). Increased cAMP concentration in the pertinent subcellular compartment(s) initiates downstream signalling pathways, primarily through activation of cAMP-dependent protein kinase (PKA), a serine/threonine kinase, but also via non-kinase effectors such as Epac, a guanine nucleotide exchange protein directly activated by cAMP [2]. Activation of the cAMP/PKA pathway in cardiomyocytes leads to an increase in intracellular Ca^2+^, which in turn activates another serine/threonine kinase, Ca^2+^/calmodulin-dependent protein kinase II (CaMKII) [3]. Recent evidence suggests that β-AR stimulation additionally modulates the activity of phosphatases, which counteract protein kinase activity by dephosphorylation of proteins. The underlying regulatory mechanisms are not fully understood but may include phosphorylation of the inhibitory protein of protein phosphatase 1 (PP1) [4] and phosphorylation [5] or translocation [6] of regulatory subunits of protein phosphatase 2A (PP2A). The dynamic phosphorylation and dephosphorylation of proteins participating in excitation–contraction coupling, cardiac metabolism and gene expression facilitate and regulate both acute and longer-term cardiac responses elicited by β-AR stimulation.

To date, investigation of cardiac protein phosphorylation events downstream of β-AR stimulation has relied primarily on treatment of isolated hearts or cardiomyocytes with β-AR agonists, followed by immunoblotting with antibodies raised against phosphorylated residues in specific proteins of interest [7,8,9,10,11]. A powerful contemporary alternative is mass spectrometry (MS)-based phosphoproteomics, which allows comparative analysis of a broad spectrum of protein phosphorylation changes in response to a given stimulus. In that context, the application of stable isotope labelling by amino acids in cell culture (SILAC) technology adds further stringency, by allowing the analysis of relative (phospho)proteomic changes in response to distinct stimuli in a quantitative manner [12]. In recent years, SILAC technology has been extended to in vivo animal models, by labelling mice with ^13^C_6_ lysine (Lys6) through the use of a lysine-free diet supplemented with Lys6 [13,14]. In a “spike-in” SILAC (or SuperSILAC) approach [15,16], protein samples from the tissue or organ of interest from Lys6 mice serve as an internal standard for quantitative (phospho)proteomic analysis of unlabelled samples from experimental mice subjected to different interventions.

**Figure 1:**
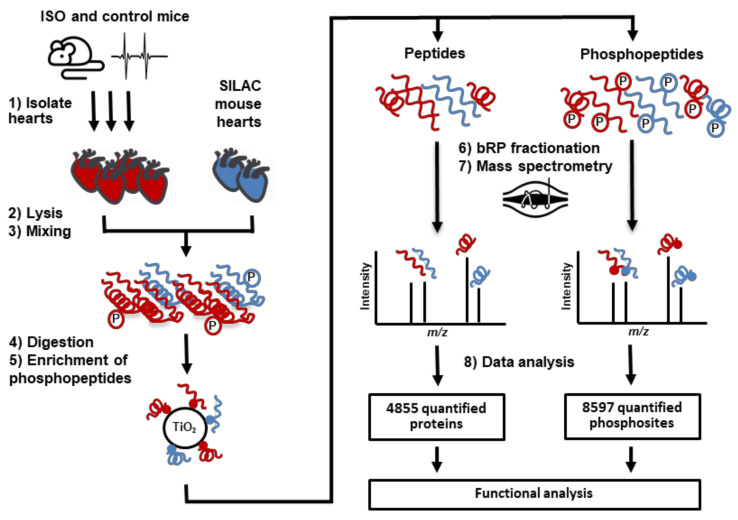
Mass spectrometry-based workflow for comprehensive analysis of protein phosphorylation in ISO-stimulated mouse hearts. Following standardization with ^13^C_6_-Lys-SILAC mouse hearts (1–3), phosphopeptides produced by tryptic digestion (4) are enriched using titanium dioxide (TiO_2_) (5), fractionated (6) and analysed by high-resolution mass spectrometry (7). In addition to differential phosphorylation analysis, nonphosphorylated peptides are employed to test for changes in protein expression (8).

Here, we utilised Super-SILAC mouse technology to perform a quantitative, comparative analysis of the cardiac phosphoproteome in mice acutely treated in vivo with either saline or isoprenaline (ISO), a nonselective β-AR agonist. Using a customized mass spectrometry-based workflow (Figure 1), our study identified hundreds of phosphosites that are significantly regulated by β-AR stimulation, including both novel phosphoproteins and phosphosites that have not been previously identified as downstream effectors of β-AR stimulation and established downstream components of cAMP/PKA and CaMKII signalling pathways. Interestingly, our study revealed both up- and down-phosphorylation in comparable numbers, indicative of an intricate signalling network activated in response to β-AR stimulation.

## 2. Results

### 2.1. Regulation of Protein Expression in Response to Acute β-AR Stimulation

**Figure 2:**
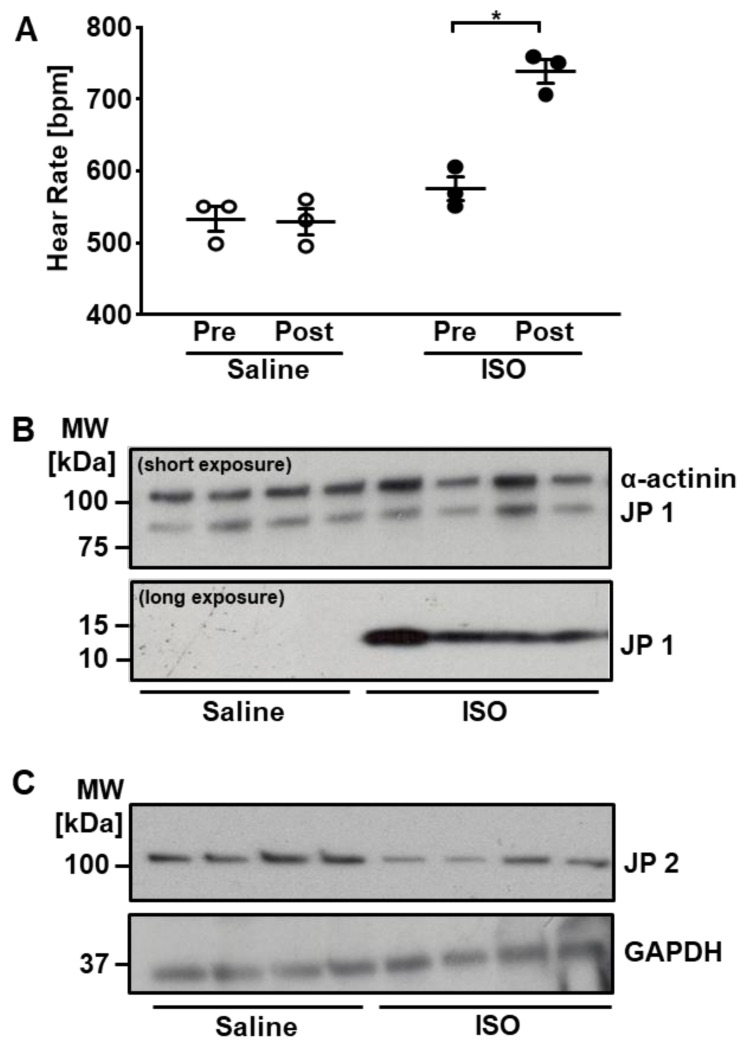
Isoprenaline (ISO) increases heart rate and triggers junctophilin 1 fragmentation. (**A**) ECG recording shows significant heart rate increase in *wt* mice (* *p* ≤ 0.05). (**B**) Expression of JP1 in ventricular tissue of mice that received ISO vs. saline. Detection by immunoblotting with an antibody directed towards the mid-region of JP1 (*n* = 4). (**C**) Corresponding expression pattern of JP2 (*n* = 4).

We administered ISO, a synthetic β-AR agonist that targets both β1-AR and β2-AR subtypes, systemically to mice, to study protein phosphorylation events induced by the β-adrenergic component of physiological sympathetic stimulation (Figure 1). The effectiveness of the acute β-AR stimulation protocol was tested by monitoring the ECG before and after injection, which revealed a significant increase in heart rate after ISO but not saline injection (Figure 2A). We first performed SILAC-based protein expression profiling in heart tissue obtained from mice that received ISO and control (CON) mice that received saline. We identified 4855 proteins at an FDR of 1% (Supplementary Table S1). We compared the protein expression profiles in both groups to exclude the possibility of major changes in protein expression that may interfere with the quantification of changes in protein phosphorylation. No significant change in protein abundance was observed following acute β-AR stimulation (Supplementary Figure S1).

### 2.2. Cardiac Expression and ISO-Induced Fragmentation of Junctophilin-1

In analysing the proteome of the mouse ventricular tissue samples used in our study, we identified unique peptides of junctophilin-1 (JP1), albeit at around a 50-fold lower concentration than JP2 as estimated by MS intensity values (Supplementary Figure S2). Previous studies on junctophilins have focused on JP2 as the principal cardiac isoform, with JP1 found to be abundant in skeletal muscle [17,18,19]. The structures of JP1 and JP2 are highly conserved across mammalian species, with the proteins observed to run on SDS-PAGE at ∼90 and ∼100 kDa, respectively [18,19,20]. To confirm JP1 expression in the heart, we performed immunoblot analysis using a JP1-specific antibody directed towards the mid-region of JP1, which is able to detect both the full-length protein and shorter JP1 fragments, which were previously reported to arise from calcium-induced, C-terminal cleavage (Figure 2B) [17,18,21]. To determine if acute β-AR stimulation impacts on cardiac JP1 protein integrity, we investigated JP1 expression in heart tissue obtained from mice that received ISO and CON mice. JP1 protein was detected in mouse ventricular tissue, at the expected molecular weight of 90 kDa. In addition, acute β-AR stimulation with ISO led to the appearance of a 15 kDa band that was not visible in cardiac tissue of CON mice. The 90 kDa intact protein was the abundant species, with the 15 kDa fragment only detected following prolonged autoradiographic exposure.

We assessed in parallel the expression of JP2, previously considered to be the predominant junctophilin isoform in the heart (Figure 2C). An antibody directed towards the C-terminal of JP2 detected the full-length protein at the expected molecular weight of 100 kDa. The abundance of full-length JP2 was reduced in response to ISO, as previously reported to occur under conditions of elevated calcium [18].

### 2.3. Regulation of Cardiac Protein Phosphorylation in Response to Acute β-AR Stimulation

Next, we analysed the enriched phosphopeptide samples obtained from ventricular tissue by mass spectrometry (Figure 1). We identified and quantified 8597 class I phosphosites, corresponding to 10,164 phospho-events from 2975 proteins that were quantified (Supplementary Table S2). Correlation analyses confirmed reproducibility of quantified phospho-events among biological replicates within each group, with an average Pearson correlation coefficient of 0.8 (Supplementary Figure S3).

**Figure 3:**
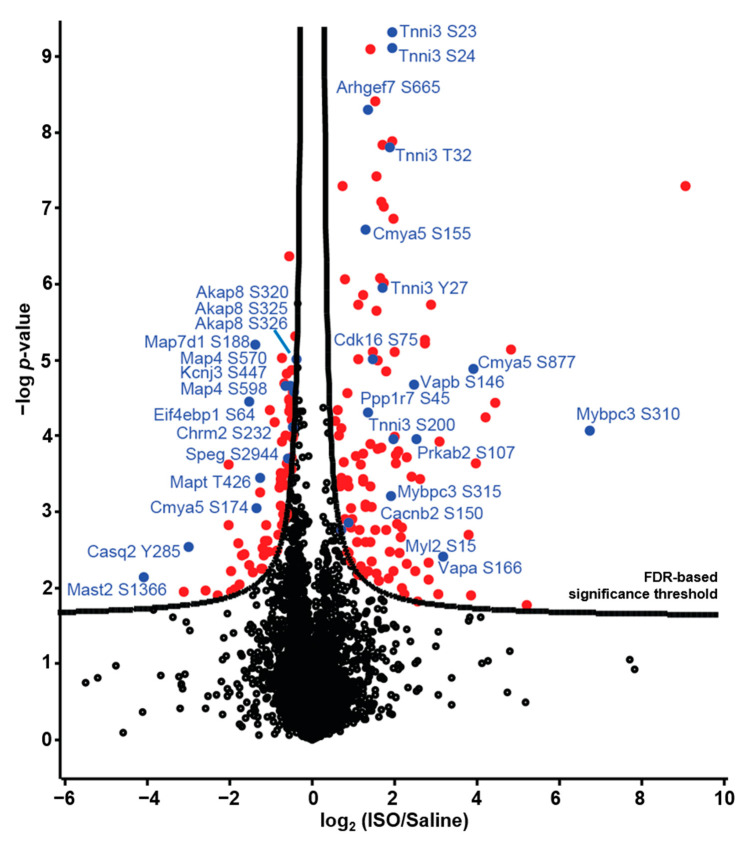
Differential protein phosphorylation in mice that received ISO versus saline. Phosphopeptides with significantly differential phosphorylation levels are marked in red; phosphopeptides pertaining to proteins of interest are marked in blue and labelled.

To determine changes in cardiac protein phosphorylation induced by acute β-AR stimulation, we compared phosphopeptide intensities in left ventricular samples from mice that received ISO and control (CON) mice that received saline (Figure 3).

We identified 197 phospho-events from 143 different proteins that displayed a statistically significant increase or decrease in phosphorylation in mice that received ISO, relative to saline controls (Figure 3). Of these, 77 phospho-events (39%) displayed decreased phosphorylation in response to ISO. Among the remaining 120 phospho-events that displayed increased phosphorylation, there were several sites (such as S15 in MLC-2 and S110 of Cdk16) that have not previously been described as downstream components of β-AR signalling, in addition to others that are well-established substrates of PKA downstream of β-AR stimulation, such as S23/24 in cTnI [22] and S302 in cMyBPC [23] The protein phospho-events that represent increased or decreased phosphorylation in response to ISO are listed in Supplementary Tables S3 and S4, respectively.

**Figure 4:**
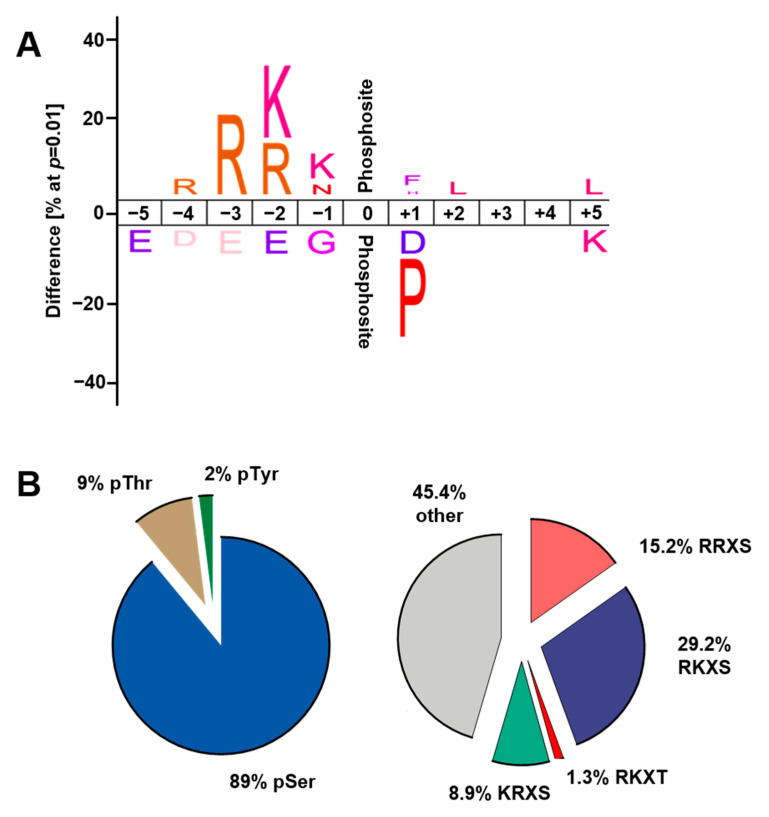
Diversity and characteristics of phosphorylation sites that displayed increased phosphorylation in response to ISO. (**A**) Motif analysis of phosphorylation sites with increased phosphorylation in response to ISO. (**B**) Distribution of phosphorylated amino acids and defined motifs for phosphorylation sites with increased phosphorylation in response to ISO.

To investigate the diversity and potential signature of cardiac protein phosphorylation events resulting from acute β-AR stimulation, we analysed the amino acid sequences flanking phosphorylation sites that displayed significantly increased phosphorylation in response to ISO. Over- or under-representation of amino acids surrounding the upregulated phosphorylation sites was determined by comparison with nonregulated phosphorylation sites. This analysis revealed overrepresentation of arginine (R) and lysine (K) in the −2 position and R in the −3 position, as well as underrepresentation of proline (P) in the +1 position (Figure 4A). The consensus motif that arises is largely consistent with that for PKA substrates [24], suggesting that increased protein phosphorylation in response to acute β-AR stimulation arises primarily from increased PKA activity. Nevertheless, there was considerable diversity among the phosphorylation sites that displayed increased phosphorylation in response to β-AR stimulation (Figure 4B). The predominant phosphoacceptor residue was serine (S, 89%), with increased phosphorylation observed also at threonine (T, 9%) and tyrosine (Y, 2%). More than half (54%) of the phosphopeptides that displayed increased phosphorylation at S or T in response to ISO conformed to the PKA consensus motif [R/K][R/K/X]X[pS/pT]. Interestingly, however, the remaining 46% of the phosphopeptides that displayed increased phosphorylation at S or T in response to ISO did not conform to the PKA consensus motif, suggesting that pathways other than direct PKA-mediated phosphorylation were involved.

To explore whether phosphopeptides that displayed altered phosphorylation in response to ISO resided in networks of proteins that are associated with certain cellular functions, we performed pathway enrichment analyses using the STRING [25] and Gene Ontology (GO) [26] databases. STRING analysis provided an intricate network of highly confident, direct and indirect functional interactions between proteins involved in key cellular processes such as myofilament function and calcium handling (e.g., cTnI, calsequestrin, titin), protein localisation and compartmentation (e.g., Ankyrin B, Sorbin and SH3 domain-containing protein 2), all of which displayed altered phosphorylation in response to ISO (Supplementary Figure S4).

**Figure 5:**
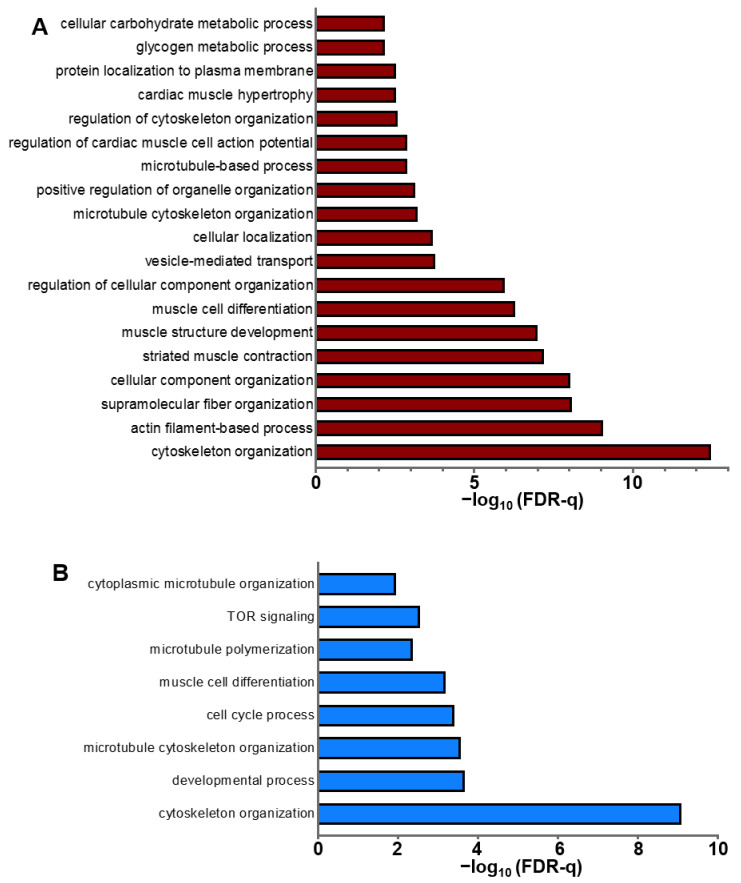
Functional enrichment analysis of proteins found to be phosphoregulated by acute β-AR stimulation. (**A**) enriched gene ontology (GO) terms for biological processes regulated by altered protein phosphorylation in response to ISO (**B**) enriched GO terms for biological processes regulated by reduced protein phosphorylation in response to ISO.

Further functional analysis of enriched GO terms highlighted important biological processes that are likely to be regulated by altered protein phosphorylation arising from β-AR stimulation, including not only those associated with electrical impulse generation/conduction and muscle contraction, but also those associated with protein localisation to subcellular compartments, muscle tissue morphogenesis, cardiomyocyte growth and metabolism (Figure 5A). We also analysed the enriched GO terms in the ranked list of a subset of proteins that only displayed decreased phosphorylation with ISO, which highlighted microtubule and cytoskeleton organisation as the most prominent processes regulated by protein dephosphorylation in response to acute β-AR stimulation with ISO (Figure 5B).

Among the individual proteins that displayed altered phosphorylation in response to ISO, we observed an overrepresentation of the ventricular isoform of myosin light chain 2 (MLC-2v) and cardiac troponin I (cTnI) within the GO terms related to myofilament contraction, and AMP activated kinase (AMPK) β2 subunit within those related to metabolism.

### 2.4. ISO-Induced Phosphorylation of AMPK β2 Subunit

**Figure 6:**
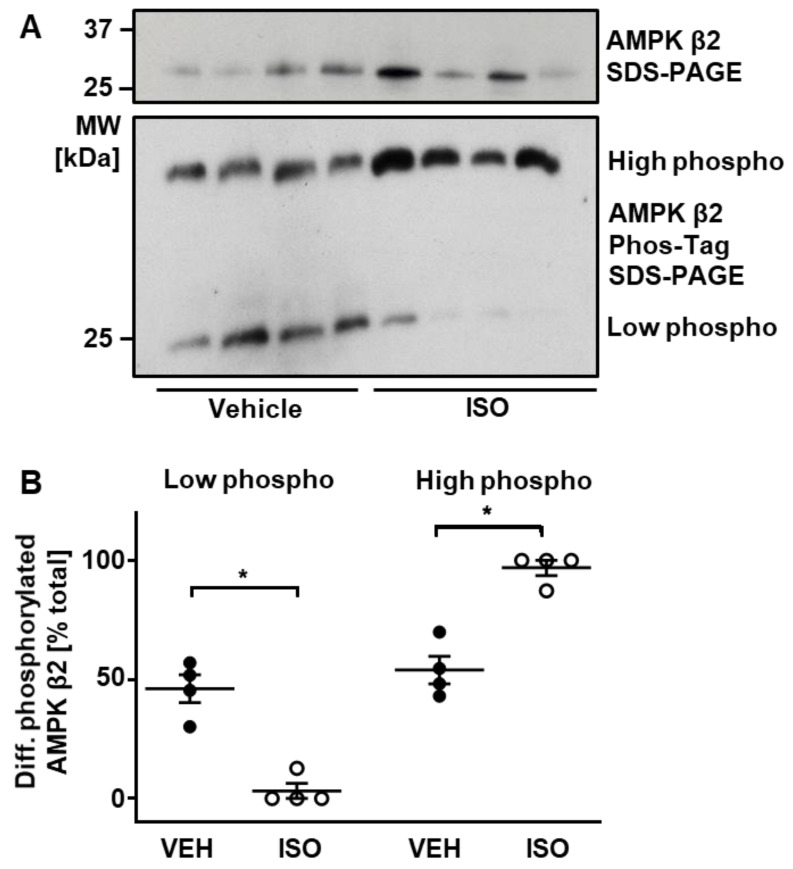
Acute β-AR stimulation increases AMPK β2-subunit phosphorylation. (**A**) Abundance of differentially phosphorylated species of AMPK β2 subunit, as assessed by immunoblotting (upper panel) and phosphate affinity (Phos-tag^TM^) SDS-PAGE (lower panel). (**B**) Significance analysis of Phos-tag^TM^ results depicted in (A). Relative abundance of species with low and high phosphorylation are expressed as a percentage of total AMPK β2, which is the sum of the signals in each lane. Lines show mean ± SE (*n* = 4 per group); * *p* < 0.05 (one-way ANOVA with Sidak’s post hoc test).

In our analysis, we detected an ISO-induced increase in the phosphorylation of AMPK β2 subunit at S108 (Supplementary Table S1). In previous studies, the noncardiac β-subunit isoform, β1, harbouring a Ser108Ala mutation, was found to be associated with a markedly reduced AMPK catalytic activity, potentially through altered subunit interactions in the enzyme heterotrimer [27,28]. We aimed to validate by an independent method that acute β-AR stimulation with ISO regulates AMPK β2-subunit phosphorylation in the heart, by using phosTAG phosphate affinity SDS PAGE electrophoresis followed by immunoblot analysis using an antibody specific for AMPK β2 subunit. As shown in Figure 6A, ventricular samples from mice that received ISO showed increased abundance of an AMPK β2 moiety displaying higher phosphorylation, with a reciprocal reduction in the abundance of the protein at a lower phosphorylation status, providing additional evidence that acute β-AR stimulation does indeed induce increased AMPK β2-subunit phosphorylation. To confirm that the two bands detected in Figure 6 represent differentially phosphorylated species of AMPK β2 subunit, we treated adult rat ventricular myocytes (ARVM) with 1 µM okadaic acid (OA) for 45 min, to inhibit PP1 and PP2A activity and elevate AMPK β2 phosphorylation. As expected, we detected an additional, slow-migrating band in the ARVM sample treated with OA, which represents phosphorylated species of AMPK β2 subunit (Supplementary Figure S5).

## 3. Discussion

Our study provides an extensive characterisation of cardiac proteins that display rapid changes in phosphorylation following β-adrenergic stimulation, the primary mechanism through which cardiac function is regulated by the sympathetic nervous system. The data reveal many novel phosphoprotein members of the signalling networks downstream of β-ARs, as well as providing evidence for both the cardiac expression and ISO-induced fragmentation of JP1.

### 3.1. Cardiac Expression and Fragmentation of JP1

Our analyses of protein expression revealed the expression of JP1 in the heart. Junctophilins are structural proteins that in cardiac myocytes hold the L-type Ca^2+^ channel and the ryanodine receptor in close proximity, and as such, play an important role in excitation–contraction (E–C) coupling [18] The literature to date suggests that JP2 is the only junctophilin isoform expressed in cardiac and smooth muscle, with JP1 being abundant in skeletal muscle. In response to adrenergic stress and elevated calcium, both JP1 and JP2 have been shown to undergo cleavage, producing lower-molecular-weight fragments [21]. In cardiac myocytes, such cleavage of JP2 is thought to result in disintegration of the E–C coupling machinery and associated Ca^2+^ handling defects [18,19]. This is the first study to confirm the expression of JP1 protein in heart tissue and to reveal fragmentation of JP1 in response to ISO. In the heart, stress-induced proteolysis of JP2 was also shown to liberate an N-terminal fragment (JP2NT) that was imported into the nucleus, where it was proposed to play an antihypertrophic role by suppressing the transcriptional activity of MEF2 [22]. The role of JP1 fragments that appear to be produced following acute β-AR stimulation in the heart remains to be investigated.

### 3.2. Cardiac Protein Phosphorylation

Our extensive dataset of 197 phosphopeptides from 143 different proteins that displayed altered phosphorylation in response to ISO markedly expands the number of known phosphoprotein targets of β-AR stimulation.

The cardiac contractile response to β-AR stimulation depends on the phosphoregulation of several myofilament proteins regulating actin–myosin cross-bridge formation, as well as that of Ca^2+^-regulatory proteins such as phospholamban and L-type Ca^2+^ channels [29]. PKA-mediated phosphorylation of cardiac troponin I (cTnI) at S23/24 in response to β-AR stimulation elicits a lusitropic effect via a reduction in myofilament calcium sensitivity, augmenting relaxation and accelerating cross-bridge kinetics [23,30,31,32]. In our phosphoproteomic analysis, increased phosphorylation of cTnI at S23/24 was found to be the top ranked phosphorylation event in response to β-AR stimulation. Interestingly, in addition to these well-characterised sites, we also detected an ISO-induced increase in the phosphorylation of cTnI at Y27 and S200, sites that have not been previously described as targets of β-AR stimulation. The Y27 and S200 residues of cTnI in mice (equivalent to Y26 and S199, respectively, in humans) were first identified as phosphoacceptor sites in a quantitative phosphoproteomics screening of failing and nonfailing hearts, where phosphorylation of Y27 was found to be reduced in failing hearts [33]. In further studies, it was found that phosphorylation of Y27 leads to reduced Ca^2+^ sensitivity of the myofilaments and accelerated thin filament deactivation, to an extent similar to that elicited by dual phosphorylation of S23/24 [34]. In other pertinent work, phosphorylation of cTnI at S200 was reported to increase myofilament Ca^2+^ sensitivity and regulate susceptibility to calpain-induced proteolysis [35]. Our data identify the cTnI residues Y27 and S200 as novel phosphorylation targets in the β-adrenergic receptor signalling pathway. Notably, the magnitude of the ISO-induced increase in Y27 and S200 phosphorylation was similar to that of S23/24. Whilst previous evidence suggests that phosphorylation of cTnI at Y27 and S200 may impact myofilament function, the implications of such phosphorylation for the regulation of cardiac function in vivo remain to be investigated.

### 3.3. Kinases and Phosphatases Displaying Altered Phosphorylation in Response to ISO

Among the proteins that displayed altered phosphorylation in response to β-adrenergic stimulation were eight protein kinases and two protein phosphatases. We detected increased phosphorylation of CDK16 (also known as Pctaire 1) at S110, which was previously shown to enhance kinase activity when phosphorylated by PKA in the brain [36]. CDK16 is expressed in several tissues, most abundantly in the brain, where it is believed to play a role in neurotransmitter release [37]. In addition, we detected a significant increase in the phosphorylation of the AMP-activated protein kinase (AMPK) β2 subunit at S108. This observation was corroborated by phos-Tag SDS-PAGE and immunoblotting, which also indicated increased phosphorylation in response to ISO. AMPK is activated by increases in AMP:ATP ratio, and modulates cellular metabolism by switching on pathways that produce ATP [38]. The enzyme is a heterotrimer composed of a catalytic α subunit and noncatalytic β and γ subunits. Whilst most studies to date have focused on the β1 subunit, as this is the most common β isoform in most cell types, β2 is believed to be the dominant isoform in cardiac and skeletal muscle [39,40]. The β1 and β2 isoforms differ in their first 65 amino acids while the remainder of the sequence, including the S108 phosphorylation site of interest, is conserved. β1 subunit harbouring a Ser108Ala mutation was found to result in a 60% decrease in the catalytic activity of AMPK and a four-fold reduction in its affinity for activation by AMP [27,28]. AMPK activation following exercise and β-AR stimulation with adrenaline has been previously demonstrated in rat adipocytes [41]. However, β-AR-induced AMPK activation has not been demonstrated in the heart, and the role of β-subunit phosphorylation in any such activation remains to be explored.

### 3.4. ISO-Induced Protein Dephosphorylation

β-AR stimulation is thought to elicit physiological responses principally via phosphorylation of key proteins by downstream kinases PKA and CaMKII. Although the majority of studies on β-AR signalling have focused on kinase-mediated phosphorylation events, a small number of studies have reported dephosphorylation of proteins by protein phosphatases in response to acute β-AR stimulation in various tissues [42,43,44,45]. In the heart, we recently reported that acute ISO stimulation leads to PP2A-mediated dephosphorylation and nuclear accumulation of HDAC5, where HDAC5 then inhibits the prohypertrophic transcription factor MEF2 [46]. In addition, an earlier study used a lysate microarray approach to determine ISO-induced changes in phosphorylation state of a set of proteins in mouse embryonic fibroblasts, and reported that several proteins were dephosphorylated in response to ISO [44]. Our phosphoproteomic analysis of cardiac tissue identified a group of 77 proteins that displayed reduced phosphorylation in response to ISO stimulation, among which there was overrepresentation of microtubule-associated proteins (MAPs) when analysed for enrichment of GO terms. The observed reductions in protein phosphorylation at pertinent sites may arise from enhanced dephosphorylation by phosphatases and/or reduced phosphorylation by kinases, which themselves may arise from altered catalytic activity and/or modified substrate-targeting in response to β-adrenergic stimulation. The role of MAP phosphorylation in regulating cardiac function in response to neurohormonal stimuli is poorly understood. This is the first report of phosphoregulation of MAPs in the context of β-adrenergic signalling. In light of the evidence that MAP phosphorylation induces cardiomyocyte apoptosis [47] and drives pathological cardiac remodelling [48], the question of whether reduced MAP phosphorylation is a protective mechanism against such detrimental effects in response to acute β-AR stimulation warrants further investigation.

### 3.5. Study Limitations

For pragmatic reasons, we used only male mice (to avoid potential variances that might arise from mixing samples from both genders and the prohibitive cost of separate analyses of hearts from male and female mice) and at a relatively young age of normal health (since our focus was on revealing potential molecular mechanisms of healthy physiological responses). Determination of potential impacts of gender, ageing and pathology on the responses that our study has revealed requires additional exploration.

## 4. Materials and Methods

Male mice maintained on a wild-type C57BL6N background at St Thomas’ Hospital (London, UK) were used in this study at 10 weeks of age and (25 ± 5) g of body weight. Animal experiments were conducted in accordance with the Home Office Guidance on the Operation of Animals (Scientific Procedures) Act 1986, published by Her Majesty’s Stationary Office, London.

### 4.1. Preparation of Mouse Tissue Samples

Mice were anaesthetized with 1.5% isoflurane. Core body temperature was maintained at 37 °C for the entire experimental procedure. Heart rate was recorded during injection of pharmacological agents. After baseline stabilization of heart rate, 0.1 mg/kg body weight dose of ISO dissolved in sterile saline (0.9% NaCl) or an equivalent volume of sterile saline was administered by intraperitoneal injection (*n* = 3 per group). Two minutes after injection, the thorax was rapidly opened, the heart was explanted, quickly rinsed in ice-cold saline and ventricular tissue was snap-frozen in liquid N_2_.

Heavy lysine (^13^C_6_-Lys)-labelled mouse hearts from two 10-week-old female mice were purchased from Silantes (Munich, Germany). Tissues were frozen in liquid nitrogen and stored at −80 °C. Frozen tissues were dissolved in a lysis buffer containing 4% SDS, 0.1 M Tris-HCL, pH 8.0 supplementing with 1x Halt Protease and Phosphatase Inhibitor Cocktail (Thermo Fisher Scientific, Dreieich, Germany), followed by 8 min (6.5 M/s, 20 s on, 1 min on ice) of homogenization using a bead mill homogenizer (FastPrep-24, MP Biomedicals, Irvine, CA, U.S.A.). The crude extract was clarified by centrifugation at 14,000× *g* for 15 min, followed by 15 min (4 °C, 15 s on, 15 s off) of sonication using a Bioruptor and final clarification by centrifugation at 14,000× *g* for 15 min. The yield of the extracted protein was measured using BCA test (Pierce BCA Protein Assay, Thermo Fisher Scientific). Purified protein from the SILAC-labelled mouse hearts was divided into equal aliquots of 1 mg and stored at −80 °C for further use.

### 4.2. Protein Extraction and Digestion

Mouse protein samples were combined with protein from the ^13^C_6_-Lys-SILAC mouse heart standard at a 2:1 ratio. Protein mixtures were reduced and alkylated with 10 mM tris(2-carboxyethyl)phosphine (TCEP) and 20 mM iodoacetamide at 37 °C for 60 min, in the dark. Three volumes of cold (−20 °C) acetone were added to precipitate the proteins in solution at −20 °C overnight. Samples were centrifuged for 10 min at 14,000× *g* and supernatant was decanted without disturbing the protein pellet. The pellet was resuspended in 0.1% Rapigest in 50 mM TEAB. Lysyl Endopeptidase (Mass Spectrometry Grade Lys-C, Wako, Neuss, Germany) was added at an enzyme-to-protein ratio of 1:100 and digested at 37 °C, overnight. Acidification was performed after trypsin digestion to make sure the pH was less than 3.0, and the solution was incubated at 37 °C for 30 min to decompose the Rapigest completely. Samples were centrifuged for 10 min at 14,000× *g* and supernatant was collected. Then, 30 μg aliquots of digested peptides from each mixed sample were used for global proteome analysis and the remaining portion was used for global phosphoproteome analysis.

### 4.3. Phosphopeptide Enrichment

Phosphopeptides were enriched directly from the purified digested peptides using a previously reported method. A total of 17 peptide solutions were dried in a SpeedVac concentrator and resuspended in an incubation buffer containing 5% glycerol in 80% acetonitrile (ACN), 5% TFA. TiO_2_ beads (10 μm; GL Sciences, Tokyo, Japan) at a 1:8 peptide-to-bead ratio were weighed and washed three times in 1 mL of 60% ACN, 0.1% TFA, 1 mL of 80% ACN, 5% TFA and 1 mL incubation buffer sequentially. The resuspended peptides were then incubated with TiO_2_ beads with end-over-end rotation at room temperature for 20 min. The peptide concentration was maintained at about 2 mg/mL during the incubation. After incubation, TiO_2_ beads were loaded onto an empty spin column (5 μm frit; Hoefer, Holliston, MA, USA) and washed three times with incubation buffer containing 80% ACN, 5% TFA and 60% ACN, 0.1% TFA. Phosphopeptides were eluted with 1% NH_4_OH (pH ≥10.5) and acidified immediately with 10% (vol/vol) TFA, maintaining the pH below 3. The eluate was desalted on a C18 spin column before fractionation.

### 4.4. High-pH Reverse Phase Fractionation

Fractionation of the peptide and phosphopeptide mixtures was performed by reverse-phase C18 chromatography (1100 series HPLC system, Agilent, Waldbronn, Germany). A total of 30 μg of each sample was dissolved in 50 μL of mobile phase A (10 mM ammonium hydroxide in water, pH 10) and separated on a reverse-phase C18 column (XBridge C18, 3.5 μm, 300 Å, 1.0 × 150 mm, Waters, Milford, MA, U.S.A.). The elution was performed using mobile phase A and B (10 mM ammonium hydroxide in 80% ACN, pH 10) at a flow rate of 60 μL/min with a gradient (2% B, 0–5 min; 2–34% B, 5–42 min; 34–60% B, 42–50 min; 60–90% B, 50–51 min; 90–90% B, 51–56.5 min; 90–2% B, 56.5–57 min; 2–2% B, 57–64 min). Peptides were detected at 214 nm, and 58 fractions were collected in a time-based mode from 6 to 64 min. Fractions were finally grouped into 18 global peptide samples and 14 enriched phosphopeptide samples using staggered pooling schemes [49].

### 4.5. Mass Spectrometric Analysis

Peptide fractions were analysed on a hybrid quadruple-Orbitrap mass spectrometer (Q Exactive HF, Thermo Fisher Scientific) coupled to a nanoflow liquid chromatography system (UltiMate 3000 UHPLC, Dionex, Thermo Fisher Scientific). Dried samples were redissolved in 2% ACN/0.1% formic acid (FA) and centrifuged for 14,000× *g* for 10 min to remove particles prior to injection. Peptides were preconcentrated and desalted on a trap column (PepMap 100 C18, 5 μm, 0.3 × 5 mm, Thermo Fisher Scientific) at 10 μL/min in loading buffer (2% (vol/vol) ACN, 0.1% FA). Peptides were separated on a self-made capillary column (ReproSil-Pur 120 C18-AQ, 1.9 μm, 300 × 0.075 mm, Dr. Maisch, Ammerbuch, Germany) using a 90 min linear gradient from 8% to 36% buffer B (0.1% FA in 80% (*v*/*v*) ACN) versus a decreasing concentration of buffer A (0.1% FA) at a flow rate of 300 nL/min.

For protein expression analysis, the mass spectrometer was operated using a top 20 data-dependent acquisition (DDA) method using higher-energy collisional dissociation (HCD) with an isolation width of 1.4 m/z and an NCE setting of 28%. MS spectra from *m*/*z* 350–1600 were acquired at a resolution setting of 120,000 FWHM (*m*/*z* 200), and MS/MS spectra at a resolution setting of 15,000. AGC target values and maximum injection times for MS and MS/MS were set to 1 × 10^6^ in 40 ms and 1 × 10^5^ in 32 ms, respectively. Fixed first mass and dynamic exclusion values were set to 110 *m*/*z* and 20 s, respectively. For phosphopeptide analysis, the same settings were used, with the exceptions of MS/MS resolution setting and maximum injection times of 30,000 at *m*/*z* 200 and 128 ms, respectively, and a dynamic exclusion duration of 25 s to improve the MS/MS spectra quality for low-abundance precursors. Each peptide fraction was analysed twice as the injection replicates.

All raw files were processed using MaxQuant software (v1.6.5.0, MPI for Biochemistry, Martinsried, Germany) [50,51]. MS/MS spectra were searched against a UniProtKB mouse database containing 62,433 protein entries (downloaded on April 2019) supplemented with 246 frequently observed contaminants via the Andromeda search engine [52]. For phosphoproteome analysis, precursor and fragment ion mass tolerances were set to 6 and 20 ppm after initial recalibration, respectively. STY phosphorylation, protein *N*-terminal acetylation, and methionine oxidation were allowed as variable modifications. Cysteine carbamidomethylation was set as a fixed modification. Enzyme specificity was set to Lys-C allowing N-terminal cleavage to proline. Minimal peptide length was set to seven amino acids, with a maximum of two missed cleavages. The false discovery rate (FDR) was set to 1% on the peptide, modification-site and protein level using a forward-and-reverse concatenated decoy database approach.

For SILAC quantitation, multiplicity was set to two for double (Lys + 0, Lys + 6) labelling. At least two ratio counts were required for peptide quantitation. Both the “match between runs” and “re-quantify” options of MaxQuant were enabled. For proteome analysis, variable modifications did not consist of STY phosphorylation, and all the other parameter settings were identical with phosphoproteome analysis.

### 4.6. Statistical Analysis

The proteinGroups.txt and phospho (STY) sites.txt tables from MaxQuant were pre-processed with Perseus (v1.6.5.0, MPI for Biochemistry) [53]. After the removal of ‘Reverse’ and ‘contaminant’ entries, the class I phosphosites with localization probability ≥0.75 were filtered. The quantitation of phosphosites is based on the phosphopeptide expression. The same phosphopeptide bearing one p-site, two p-sites or more than two p-sites may be quantified together in the sample. In order to make full use of the quantitative phosphopeptides information, we analysed the data from the phosphorylation event (phospho-event) level. The function of “Expand site table” was used to convert the phosphosites into phospho-events. Protein expression ratios from the ^13^C_6_-Lys-SILAC-labelled mouse heart standards compared to mouse protein samples were normalized to achieve the median of all quantified proteins in each replicate as 0.5. Phospho-event ratios were normalized by the median of all quantified protein expression ratios in each replicate. Two-sample *t*-test in the volcano plot was performed to filter the significantly regulated proteins and phospho-events with the Permutation-based FDR less than 0.5% and S0 (background variability) of 0.1. For quality control, multiscatter plots displaying Pearson’s correlation coefficients were made in Perseus.

Consensus motifs were determined using the IceLogo software [52]. Consensus motifs were generated from 120 phospho-events that displayed increased phosphorylation with ISO, with the complete set of identified phospho-events serving as the reference set.

The phosphoprotein association network was generated on STRING (version 11.0). Edges indicate both functional and physical protein associations and disconnected nodes (noninteracting proteins) were omitted. The minimum required interaction score was set to 0.400 (medium confidence). Enriched GO terms were extracted from the STRING analysis, with *p*-values corrected for multiple testing using the Benjamini–Hochberg procedure [25,26].

## 5. Conclusions

Our study represents the first quantitative phosphoproteomic profiling of cardiac ventricular tissue in response to acute β-AR stimulation in vivo. The novel β-AR mediated phosphorylation events identified here provide a resource for future experimentation to obtain mechanistic information not only on the acute physiological responses, but also on potential longer-term pathological outcomes associated with aberrant stimulation in the context of cardiovascular disease.

## Data Availability

The mass spectrometry proteomics data have been deposited to the ProteomeXchange Consortium via the PRIDE [54] partner repository with the dataset identifier PXD025569.

## Supplementary Materials

The following are available online at https://www.mdpi.com/article/10.3390/ijms222212584/s1, Figure S1: Differential protein expression analysis by SuperSILAC-MS in mice, Figure S2: Ranked abundance analysis of SuperSILAC-MS protein expression data, Figure S3: Reproducibility of phosphopeptide quantitation, Figure S4: STRING-DB analysis of proteins exhibiting differentially phosphorylated peptides upon ISO stimulation, Figure S5: Validation of identity of bands detected by Phos-tag SDS-PAGE and immunoblotting, Table S1: Protein Expression Data, Table S2: Phosphorylation Data, Table S3: Up-Phosphorylation, Table S4: Down-Phosphorylation.
