# Supplementary material for: Quantitative Analysis of the Cardiac Phosphoproteome in Response to Acute β-Adrenergic Receptor Stimulation In Vivo
Source: Int J Mol Sci. 2021 Nov 22;22(22):12584. doi: 10.3390/ijms222212584 (PMC8618155; doi:10.3390/ijms222212584)
Supplement: Supplementary file 1 [file ijms-22-12584-s001.zip › ijms-1385076-supplementary figures.pdf]

International Journal of
Molecular Sciences

MDPI

Article

Unbiased quantitative analysis of the cardiac phosphoproteome in response to acute β-adrenergic receptor stimulation *in vivo* – supplementary figures

Alican Güran [1†], Yanlong Ji [2,3,4†], Pan Fang [2,5], Kuan-Ting Pan [2,3,4], Henning Urlaub [2,6], Metin Avkiran [1] and Christof Lenz [2,6*]

1 School of Cardiovascular Medicine and Sciences, King's College London, British Heart Foundation Centre of Research Excellence, St Thomas' Hospital, Westminster Bridge Road, London SE1 7EH, United Kingdom
2 Bioanalytical Mass Spectrometry Group, Max Planck Institute for Biophysical Chemistry, 37077 Goettingen, Germany
3 Hematology/Oncology, Department of Medicine II, Johann Wolfgang Goethe University, 60590 Frankfurt am Main, Germany
4 Frankfurt Cancer Institute, Johann Wolfgang Goethe University, 60590 Frankfurt am Main, Germany
5 Department of Biochemistry and Molecular Biology, Soochow University Medical College, 215123 Suzhou, P. R. China
6 Department of Clinical Chemistry, University Medical Center Goettingen, 37075 Goettingen, Germany
† Both authors contributed equally to this work.
* Correspondence: Christof.lenz@med.uni-goettingen.de

Received: date; Accepted: date; Published: date

Abstract: β-adrenergic receptor (β-AR) stimulation represents a major mechanism of modulating cardiac output. In spite of its fundamental importance, its molecular basis on the level of cell signaling has not been characterised in detail yet. We employed mass spectrometry-based proteome and phosphoproteome analysis using SuperSILAC (spike-in stable isotope labelling by amino acids in cell culture) standardization to generate a comprehensive map of acute phosphoproteome changes in mice upon administration of isoprenaline (ISO), a synthetic β-AR agonist that targets both β1-AR and β2-AR subtypes. Our data describe 8597 quantitated phosphopeptides corresponding to 10164 known and novel phospho-events from 2975 proteins. 197 of these phospho-events showed significantly altered phosphorylation, indicating an intricate signaling network activated in response to β-AR stimulation. In addition, we unexpectedly detected significant cardiac expression and ISO-induced fragmentation of Junctophilin-1, a Junctophilin isoform hitherto only thought to be expressed in skeletal muscle. Data are available via ProteomeXchange with identifier PXD025569.

Keywords: phosphorylation; cell signaling; mass spectrometry; β-adrenergic receptor; SILAC

Supplementary Figure S1. Differential protein expression analysis by SuperSILAC-MS in mice

Supplementary Figure S2. Ranked abundance analysis of SuperSILAC-MS protein expression data

Supplementary Figure S3. Reproducibility of phosphopeptide quantitation

Supplementary Figure S4. STRING-DB analysis of proteins exhibiting differentially phosphorylated peptides upon ISO stimulation

Supplementary Figure S5. Validation of identity of bands detected by Phos-tag SDS-PAGE and immunoblotting

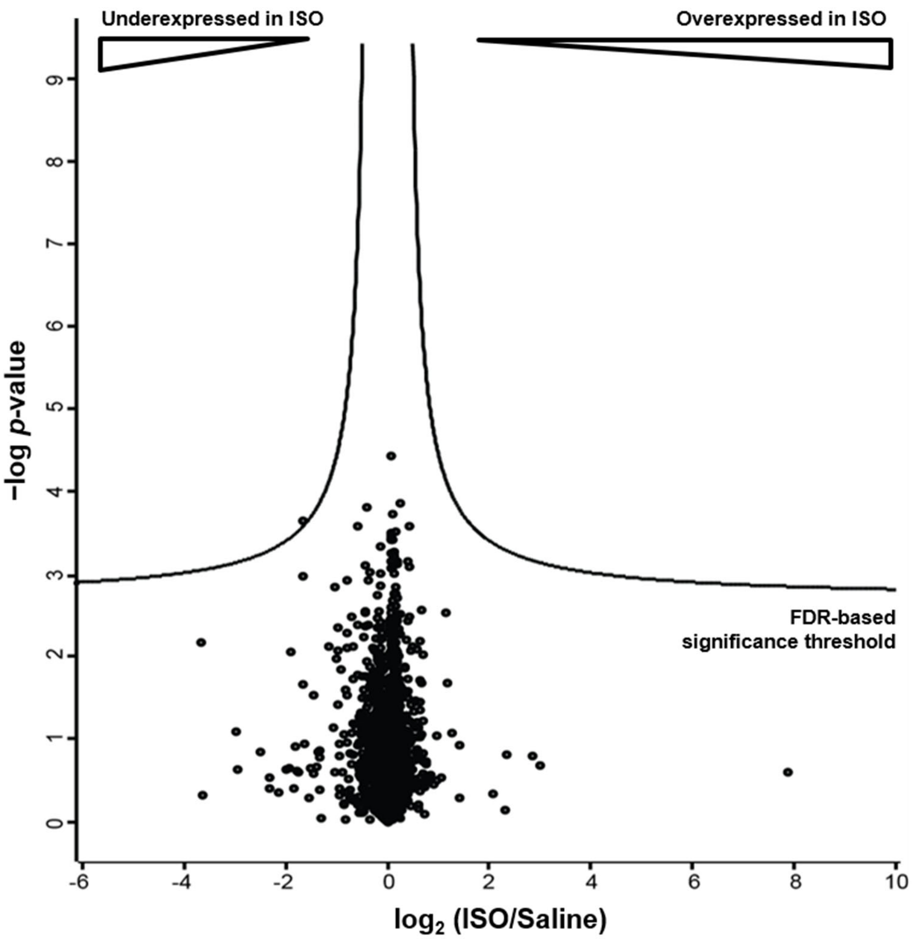

Supplementary Figure S1. Differential protein expression analysis by SuperSILAC mass spectrometry in mice that received ISO versus saline. Significance analysis shows no signs of protein abundance changes under the experimental conditions.

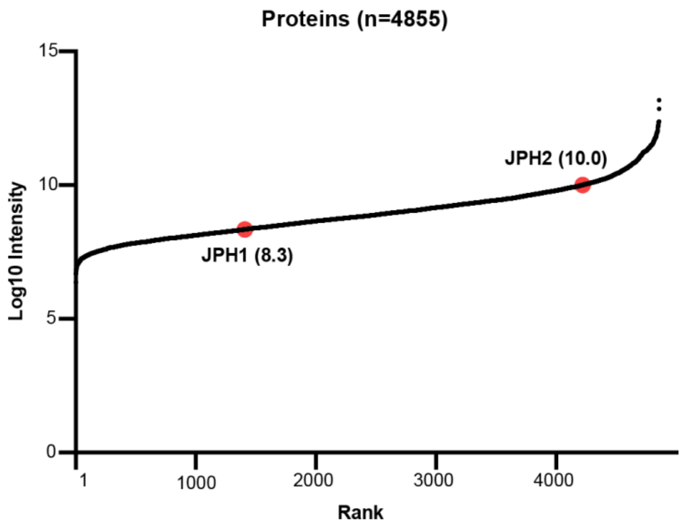

Supplementary Figure S2. Ranked abundance analysis of SuperSILAC mass spectrometry protein expression data shows significantly higher abundance for the known cardiac Junctophilin isoform JP2 (here: JPH2) than for the newly-detected JP1 (here: JPH1).

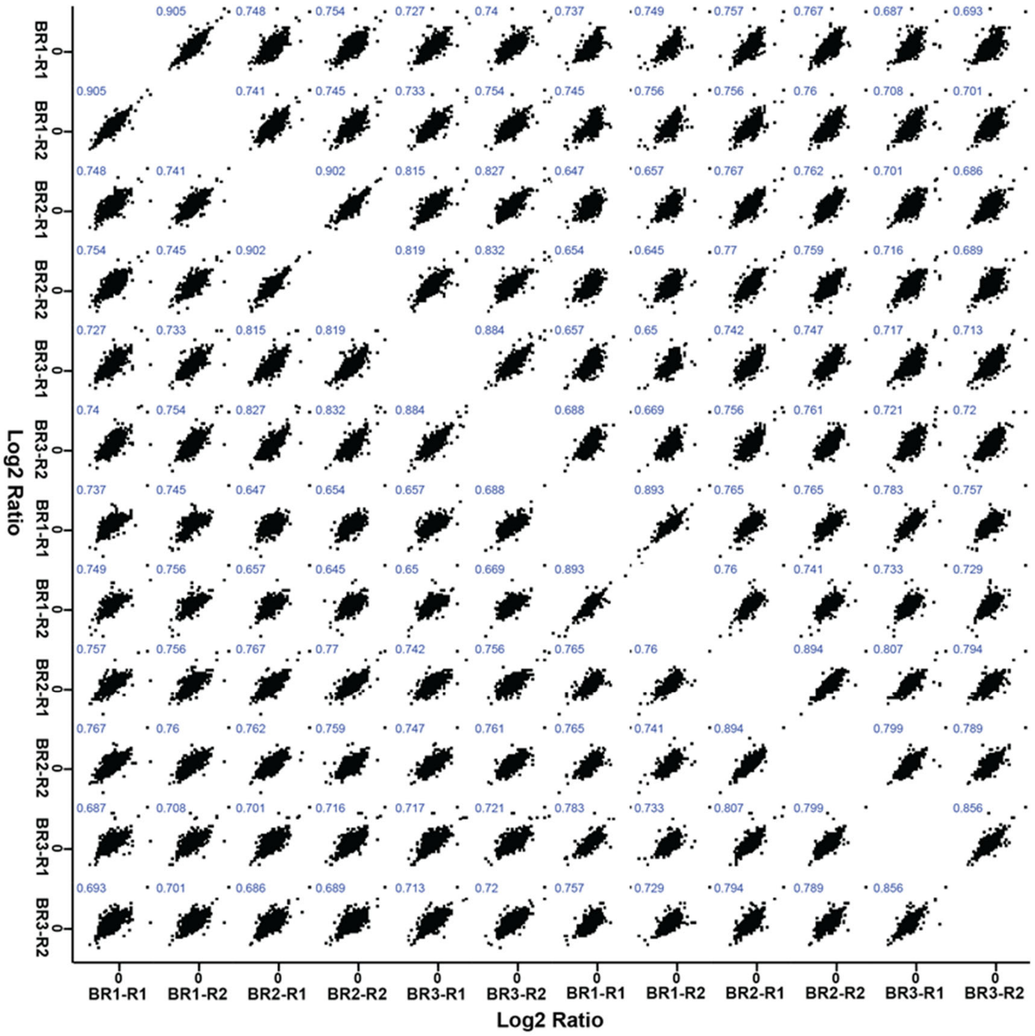

Supplementary Figure S3. Reproducibility of phosphopeptide quantitation across biological (BR1-BR3) and technical (-R1 vs –R2) replicates.

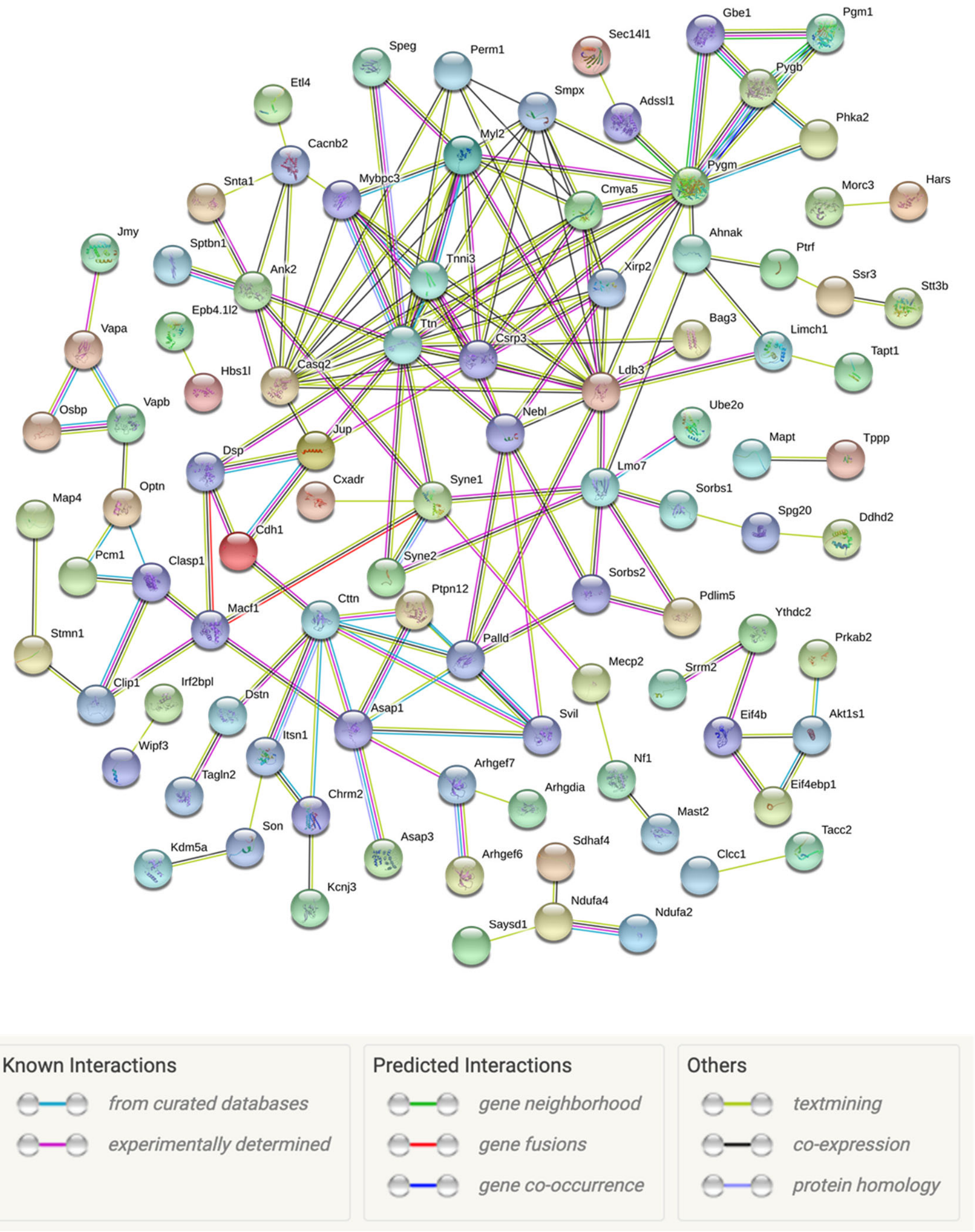

Supplementary Figure S4. STRING-DB analysis of proteins exhibiting differentially phosphorylated peptides upon ISO stimulation. Clustering of known interactions reveals Casq2, TTn, Tnni3, Csrp3, Mybpc3, Myl2, Cmya5, Ldb3 and Pygm as central hubs.

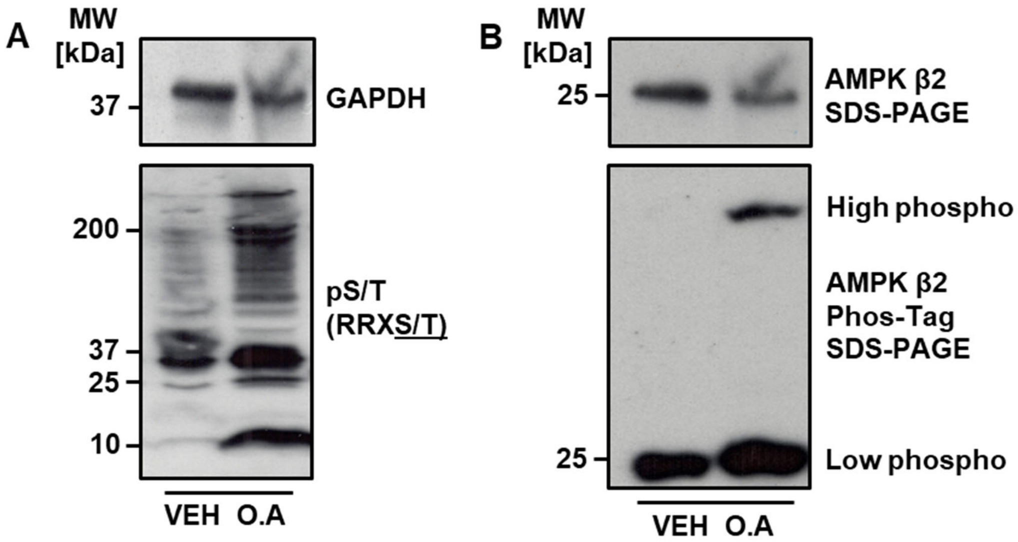

Supplementary Figure S5. Validation of identity of bands detected by Phos-tag SDS-PAGE and immunoblotting. Adult rat ventricular myocytes were treated with vehicle (DMSO) or okadaic acid (O.A) to elevate protein phosphorylation by non-selective inhibition of endogenous PP1 and PP2A activity. The appearance of a slower-migrating band in the O.A treated cells confirmed the identity of this band as the AMPK β2 subunit with increased phosphorylation, relative to the faster-migrating band.
